# Children’s Menus at Fast Food Restaurants on the Uber Eats^®^ Delivery App

**DOI:** 10.3390/foods14030434

**Published:** 2025-01-29

**Authors:** Andrea Zapata-Quiroga, João P. M. Lima, Ada Rocha, Silvana Saavedra-Clarke, Samuel Durán-Agüero

**Affiliations:** 1 Clínica Red Salud, Santiago 8320000, Chile; andrea.zapata.quiroga@gmail.com; 2 H&TRC—Health & Technology Research Center, Coimbra Health School, Polytechnic University of Coimbra, 3045-043 Coimbra, Portugal; joao.lima@estesc.ipc.pt; 3 GreenUPorto—Sustainable Agrifood Production Research Centre, 4099-002 Porto, Portugal; 4 SUScita—Research Group on Sustainability, Cities and Urban Intelligence, 3045-043 Coimbra, Portugal; 5 GreenUPorto—Sustainable Agrifood Production Research Centre, Inov4Agro, Faculty of Nutrition and Food Sciences, University of Porto, 4099-002 Porto, Portugal; adarocha@fcna.up.pt; 6 Carrera de Nutrición y Dietética, Facultad de Ciencias de la Salud, Universidad Autónoma de Chile, Providencia 7500000, Chile; saavedraclarke@gmail.com; 7 Escuela de Nutrición y Dietética, Facultad de Ciencias para el Cuidado de Salud, Sede los Leones, Universidad San Sebastian, Providencia 7500000, Chile

**Keywords:** children’s menu, obesity, food offer, digital delivery apps

## Abstract

Objectives: To evaluate the offer of children’s menus offered in fast food restaurants present in the Uber Eats delivery application through the Kids Menu Healthy Score ‘KIMEHS’ in Greater Santiago. Methods: Observational, descriptive, cross-sectional. Research in fast food restaurants present in the Uber Eats delivery app. A total of 858 restaurants were selected. The KIMEHS was used to assess menu quality. KIMEHS index and descriptive statistics were calculated. Results: 558 restaurants were evaluated through the app; 57 offered children’s menus, yielding 114 children’s menu options from 18 different municipalities. The common offer was based on fried and/or processed lean meat accompanied by French fries in 71%. Moreover, 99% of the menus assessed obtained the minimum score in the KIMEHS placing them in the ‘not healthy at all’ category. When associations were made between foods and the lowest KIMEHS score quartile, the presence of chips had the strongest association (OR; 40.36: CI95% 11.43–201.08). Conclusions: Most restaurants offer a children’s menu of low nutritional quality and poor balance, where their dishes are commonly based on fried and processed products, pointing to the urgent need for legislation on guidelines to be applied on the different actors influencing the food offered to children.

## 1. Introduction

The introduction of digital delivery applications in Chile, marked by the arrival of Uber Eats, Glovo, and Rappi in late 2017 and early 2018, revolutionized food consumption patterns, particularly in the “ordering food at home” category [1]. The COVID-19 pan-demic further accelerated this shift, with the use of digital channels for food delivery soaring from 3% in 2019 to 25% in 2021 [2]. These platforms provided not only a practical solution to minimize infection risks but also an employment avenue for many affected by travel restrictions and extended quarantines [3]. This rapid adoption reshaped food systems across production, distribution, and marketing, while transforming the economics of transportation, packaging, and storage [4].

The widespread use of delivery services has driven significant changes in consumer behavior, industry competition, and social dynamics, creating both opportunities and challenges. For instance, these platforms have enabled faster and more convenient access to meals but have also contributed to higher consumption of ultra-processed, nutrient-poor foods high in sodium, saturated fats, and sugar. Concurrently, fewer instances of home-cooked meals and shared family dining have emerged as a byproduct of this convenience-driven culture. These shifts have compounded existing public health concerns, including the rising prevalence of obesity and related chronic diseases, particularly among children [5,6].

A study in Chile indicated that Chileans feel more comfortable buying various foods through last mile applications (15%), supermarket applications (39%), and even messaging entrepreneurs such as Whatsapp (27%) [2]. Food delivery applications grew explosively in recent years, providing work to a large number of people and providing a relevant service, especially during the COVID-19 pandemic [3,7]. The Chilean user participation rate in e-commerce reached 80% by the end of 2020, and food delivery was consolidated in the food category, reaching 67% [8]. Among the most frequently requested food items, the well-known McCombo Grande Double Quarter Pounder and Papa John’s with “Arma tu pizza” are mentioned with 900,000–1,000,000,000 annual requests, according to statistical data delivered by Uber Eats. In addition, it was determined that in the year 2022, Chileans ordered French fries every day without exception. Among the most requested foods, from highest to lowest, were sushi, Chinese food, hamburgers, sandwiches, pizza, healthy food, and chicken [9].

Technology’s role in entertainment and consumption has also diminished time spent in healthier, active pursuits, further contributing to obesogenic environments. The con-vergence of these factors has intensified the childhood obesity crisis, an issue the World Health Organization (WHO) has identified as urgent and critical to address [10,11]. Dietary patterns, particularly the reliance on ultra-processed foods, are a key driver of this imbalance [11]. These products, characterized by high caloric density, low nutritional value, and attributes that encourage overconsumption, are now a major dietary cause of weight gain and chronic illnesses [12]. Because they are nutritionally unbalanced and have peculiar non-nutritional attributes that promote overconsumption, ultra-processed products are most likely the main dietary cause of weight gain and chronic diseases [13,14]. Family choices when ordering delivery are of crucial importance in the nutritional future of new generations where fast food appears to be a convenient and attractive option for those who feel pressured by work constraints and multiple occupations, who may choose to spend little time eating and who have easy access to ready-to-eat snacks and meals [15,16,17].

Research from 2015 highlighted that most children’s menus exceeded the daily recommendations for fat and sodium, with 40% providing over half of a child’s daily energy needs [18]. A multicenter study conducted in 2021 across five countries, including Chile, further underscored the limited availability of healthy options on children’s menus in shopping mall restaurants [19]. In Chile’s Los Lagos region, for example, 47.1% of children aged 4–13 consumed junk food at least once a week in 2020, with 17.1% consuming it twice or more, reflecting the high prevalence of calorically dense and nutritionally poor meals [20].

Given this context, the objective of this study is to evaluate the nutritional quality of children’s menus offered by fast food restaurants available on the Uber Eats delivery platform in Greater Santiago. Using the Kids Menu Healthy Score (KIMEHS), this research aims to shed light on the health implications of these offerings and contribute to strategies for improving dietary options for children.

## 2. Materials and Methods

### 2.1. Research Design

An observational, descriptive, cross-sectional, descriptive research study was conducted in fast food restaurants present in the Uber Eats delivery application in different districts of greater Santiago (approximately 7 million people). The selection of restaurants was carried out randomly within the application, entering as “users or customers” and repeated restaurants or fast food chains were not accepted. A central location was established in the commune where the order request was simulated and restaurants in neighboring communes (shown by the delivery application) were also considered.

### 2.2. Units of Analysis

The selected communes were classified according to the social priority index (SPI), whose first version was made in 1995 and is a composite indicator that integrates relevant aspects of communal social development such as the dimensions of income, education, and health [21]. The version used corresponds to the new 2022 update.

The Kids Menu Healthy Score “KIMEHS” was used to evaluate the quality of the menus offered by the restaurants. This tool aims to evaluate the food supply aimed at children by reviewing the menus available on the websites or on the menus displayed in the restaurants themselves. It includes 18 components, divided into seven main groups reflecting key aspects of menu quality, such as protein source, side dishes, vegetables, dessert, and beverages, as well as allergens and nutritional information. The possible score ranges from −17 to 17, with a higher score indicating greater compliance with the recommendations. The Kids Menu Healthy Score (KIMEHS) [22] is an evaluation tool developed to assess the alignment of kids’ menus with dietary recommendations for children, emphasizing the prioritization of the consumption of vegetables, cereals, starchy vegetables, pulses, fruit, fish, and water while limiting processed and red meats, as well as sugar-rich foods. The KIMEHS index was designed to provide a comprehensive assessment of menu quality, and positive points are awarded for healthy menu options, while unhealthy choices receive negative points. These points’ magnitude reflects each component’s relative impact on menu quality and health outcomes.

Protein sources are subdivided into 8 items that consider the type of protein (animal vs. plant-based), cooking method, and inclusion of processed meats. For instance, fish—valued for its high biological and sustainable protein content—is awarded 2 points, while pulses, though sustainable but lower in biological protein value, score 1.5 points. Lean meats are given 1 point due to their lower sustainability than fish or pulses, and red meats score 0.5 points because of their higher saturated fat content. Processed meats, such as sausages, nuggets, bacon, and fish fingers, incur penalties, with their presence scoring −2 points. Similarly, fried items like fried potatoes receive −2 points, while their absence is rewarded with +1 points. Side dishes are evaluated based on their nutritional composition. Common options like rice, pasta, bread, corn, and potatoes receive +1 point, while the inclusion of fried potatoes incurs a −2 point penalty. Due to insufficient information from menu descriptions, whole-grain content could not be assessed. Vegetables play a crucial role in a healthy, sustainable diet, providing essential fiber, vitamins, and minerals while supporting weight management and reducing the risk of chronic diseases such as cardiovascular conditions, certain cancers, diabetes, and obesity [23,24]. Recognizing their significance, vegetables in soups or as standalone components score +2 points, while their absence incurs a penalty of −2 points. Desserts are assessed to promote healthier choices. Menus offering fruits are awarded +2 points, while their absence incurs −2 points. Conversely, sweet desserts are penalized with −1 point for their presence, while their absence is rewarded with +1 point. Beverages are evaluated to encourage water consumption as the recommended drink. The inclusion of water scores +2 points, and its absence incurs −2 points. Sugary beverages, including soft drinks and fruit juices, receive −1 point, while their absence is rewarded with +1 point [25]. Nutritional and allergen information on menus is also considered. Each type of information scores +0.25 points if present, though no penalty is applied for its absence. The final KIMEHS [22] score is the sum of all components, ranging from −17 to +17. A negative score indicates poor menu quality with limited alignment to nutritional guidelines, whereas a higher score reflects greater adherence to dietary recommendations.

A value of 5.5 is obtained if all KIMEHS items are available, considering healthy and unhealthy options. The evaluation of the menu based on the score obtained is as follows (adaptation to Spanish): −17 to 0.5 points not at all healthy or junk food (unhealthy); from 0.5 to 5.5 points unhealthy food (moderately unhealthy); from 5.5 to 11.5 points regularly healthy food (going healthy); from 11.5 to 13.5 points healthy food (moderately healthy) and from 13.5 to 17 points very healthy food (healthy) [22].

**Figure 1:**
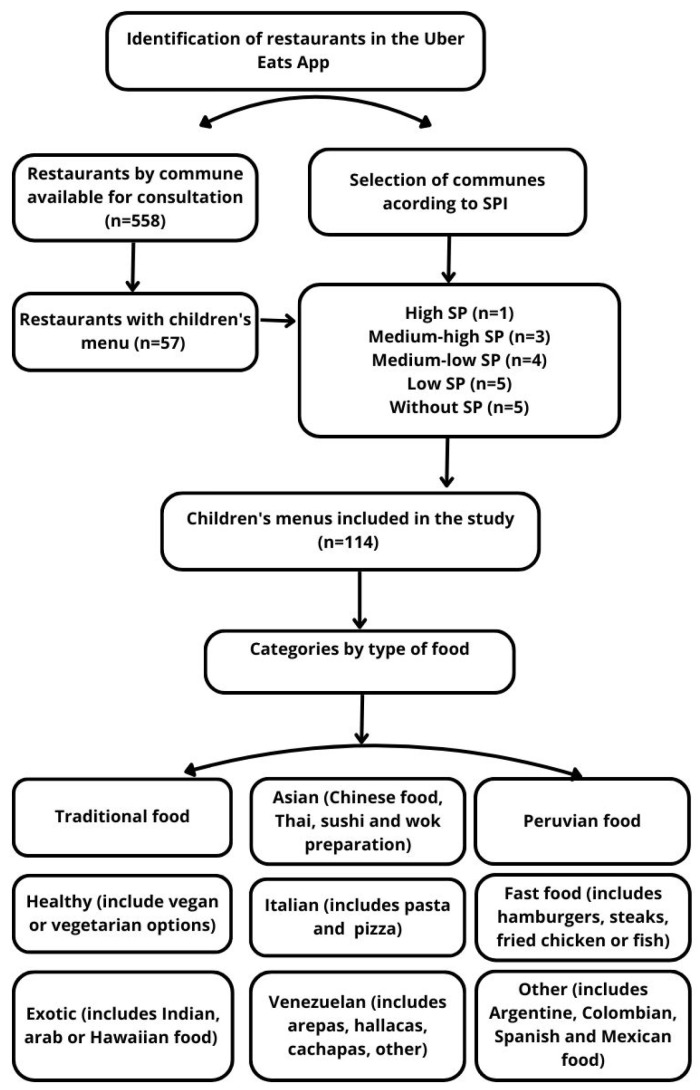
Flow chart, selection of restaurants with children’s menu, SPI, and categories by type of food, 2023.

Data collection took place between July and August 2023 and used only data publicly available in the application without accessing secondary data from the application. Google Forms was used for data collection, organized in two sections: A: location, type of restaurant, and characteristics, and B: characteristics of the children’s menu (number of options, menu items, including meat and fish options, vegetables, desserts, fruits and beverages, collectible toys, nutritional information and allergens, cost of the children’s menu). This tool was previously validated in the multicenter study conducted in 2021 Children’s Menus in Shopping [19]. For the analysis of the data, the restaurants/fast food chains were grouped by categories according to the predominant type of dish: traditional (which includes a la carte options and typical dishes), healthy (includes vegan or vegetarian options), Asian (includes Chinese food, Thai, sushi and wok preparations), Italian (includes pastas, gnocchi and pizzas), Peruvian (includes typical Peruvian gastronomy), fast food (includes hamburgers, steaks, churrascos, and fried chicken or fish), exotic (includes Indian, Arab, or Hawaiian food), Venezuelan (includes arepas, cachapas, hallacas, pabellón criollo), and others (includes typical Argentine, Colombian, Spanish, and Mexican food) (Figure 1).

### 2.3. Universe and Sample

Data from a total of 558 restaurants available for consultation in the Uber Eats application, of which 57 restaurants presented children’s menu options, were collected, obtaining a total of 114 children’s menus included in the study.

### 2.4. Data Analysis

For the statistical analysis of the data, the R Commander software package, version number 2.9-5 “Library (Rcmdr)” was used and the KIMEHS index was calculated using Microsoft Excel for Microsoft 365 MSO version 2308.

**Figure 2:**
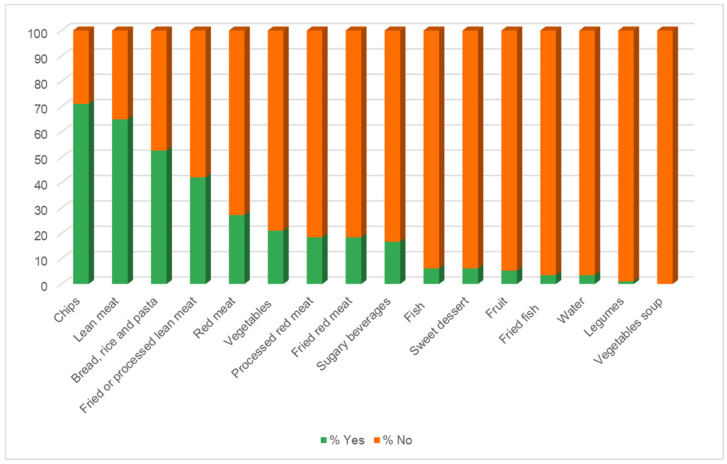
Percentage of foods present on menus.

To analyze the prices ($USD) of the menu by communes classified by IPS, Kruskal–Wallis was used since the prices of children’s menus did not have a normal distribution and also their variances were not homogeneous, ruling out the possibility of performing Anova. For the logistic regression, the children’s menu was dichotomized as follows: less healthy menus and unhealthy foods (lowest scoring quartile: −17; −8 points) and other (≥−7 points); furthermore, the presence of healthy foods was categorized as 1 and the absence as: 0. The logistic regression model was used, and the following adjustments were made: Crude model: No adjustments (model 1), and the most frequent foods were incorporated. In model 2, moderately frequent foods were added. In model 3, the foods with the lowest presence were added (following the order of Figure 2). A value of *p* < 0.05 was considered significant. To determine the association, the OR values and 95% confidence interval (95%CI) are presented. A value of *p* < 0.05 was considered significant.

## 3. Results

A total of 858 restaurants were selected, of which 57 restaurants offered children’s menus, obtaining a total of 114 children’s menu options. Regarding the number of children’s menu options, it was found that there were generally one (25.4%), four (24.5%), three (23.6%), two (17.5%), and to a lesser extent, five menu options per restaurant (8.7%).

Regarding the offer of collectible toys in children’s menus, only 8% of the menus evaluated included this offer.

The communes were selected according to their social priority index (SPI), shown in Table S1.

### 3.1. KIMEHS Components

Table 1 shows the percentage availability of the KIMEHS components in the menus evaluated, where it is observed that the common offer is fried and/or processed lean meat, generally Nuggets or breaded chicken fillets, followed by fried and processed red meat (hamburgers), and to a lesser extent, fish, approximately 50% of which is fried. Side dishes were mainly French fries (71%) and others, including pasta, rice, and bread (50.8%). Vegetables were found in 21%, mainly as sautéed, salads, and sticks. Most of the menus do not offer pulses or soups in their children’s options.

Both fruit and sweet desserts are present in smaller quantities, it is evident in the study that they generally have an additional cost in the order, and the fruit is generally found as packaged fruit puree and not as natural fruit itself. In relation to the beverage, it was also evident that in some menus, it was not included or had an additional value, but still sugary drinks lead the way. Water, when available, is an option among sugary drinks.

No children’s menu had nutritional information available and allergen information was found to be available on only two of the menus evaluated (1.75%).

Figure 2 shows the percentage presence of foods on children’s menus: chips; lean meat; bread, rice, and pasta; and fried or processed lean meat are the most prevalent foods, while water, legumes, and vegetables soup are the least prevalent, and sugar-sweetened beverages appear in the middle of the figure.

The food availability of the children’s menus of the different restaurant categories by type of food is presented in Table 2.

Red meat is mostly found in fast food and Venezuelan food (fried and processed), while lean meat is available in all categories at over 50% and is mostly used fried or processed in the exotic and Venezuelan categories. Asian and Peruvian restaurants are the only ones that offer fish on their menus (14%), which is usually fried. None of the categories listed in Table 2 have pulses available.

The categories “Other” and “Healthy” have only one children’s menu each and are therefore an exception to the rule, the latter being the only category to offer legumes in its children’s menu.

In all restaurant categories, the availability of French fries exceeds 50%, except in the Italian category. In addition, all categories offer other types of side dishes such as bread, rice, and pasta. Vegetables are found in higher percentages in the Italian and exotic categories and in smaller quantities in traditional and Asian restaurants. No category offered vegetable soups with vegetables within the children’s menu options available in the application. Fruit and sweet desserts were most frequently offered in the fast food and Peruvian categories, with sweet desserts being offered exclusively in traditional and Asian restaurants. Water was only available between 9 and 20% in fast food and exotic food restaurants. Sugar-sweetened beverages were available in all restaurants except Venezuelan restaurants.

The “Other” and “Healthy” categories, as in the previous table, are an exception to the rule because they have only one children’s menu per category.

### 3.2. KIMEHS Score

The total average of the sample of children’s menus evaluated in the MR was −6.1 KIMEHS points with a maximum score of 2 and a minimum of −12. Only four of the menus evaluated obtained a positive score with scores ranging from 0.25 to 2 points, corresponding to three restaurants, while three menus obtained a neutral score (0 points) corresponding to two restaurants. Additionally, 99% of the menus evaluated obtained the minimum score in the KIMEHS, remaining in the “unhealthy” category (n: 113), and only one children’s menu managed to obtain a positive score, even so, remaining in the “unhealthy” menu category according to the KIMEHS index classification mentioned in the methodology.

**Figure 3:**
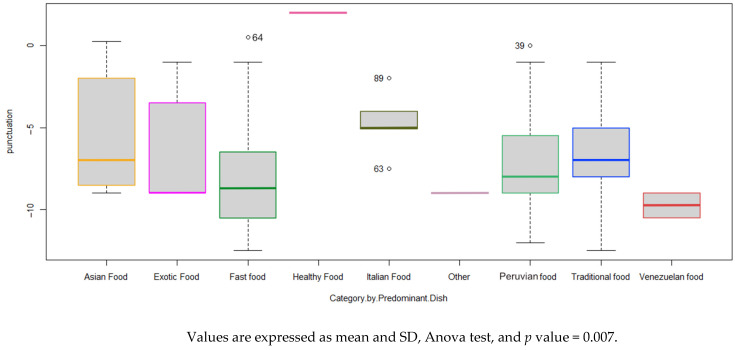
KIMEHS score according to category by type of food.

Figure 3 shows the KIMEHS score obtained according to the category of food or main dish, showing that all the restaurants analyzed in the study obtained negative or neutral scores, with the lowest scores in the fast food, traditional, Peruvian, and Venezuelan categories. On the other hand, the highest score corresponds to the healthy category (only one restaurant) with 2 points, followed by fast food, Asian, and Peruvian with neutral scores, respectively. All categories obtained negative average scores except healthy. On the other hand, the worst average score corresponds to Venezuelan food.

The KIMEHS score of children’s menus are significantly different according to main dish categories (*p* < 0.05).

### 3.3. Cost of the Children’s Menu

**Figure 4:**
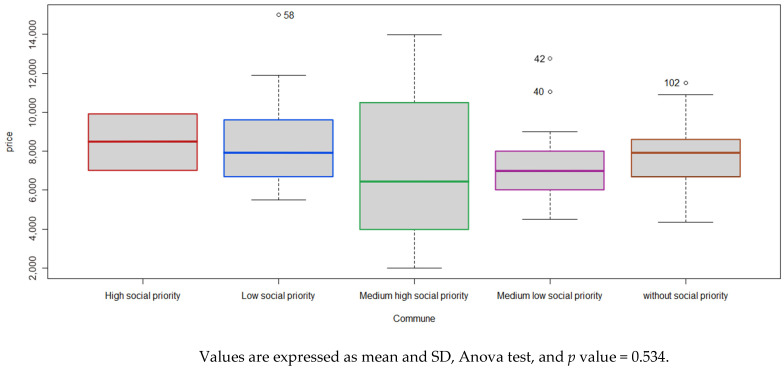
Cost of children’s menus according to communes grouped by social priority index (SPI).

According to the SPIz4 category, Figure 4 shows that the average value of the children’s menu in the high social priority category was 8.6 (SD = 1.4), medium high social priority was 7.3 (SD = 4.1), medium low social priority was 7.7 (SD = 2.5), low social priority was 8.5 (SD = 2.3), and no social priority was 7.6 (SD = 1.7). No significant differences were observed between mean values of children’s menus among districts distributed according to SPI.

**Figure 5:**
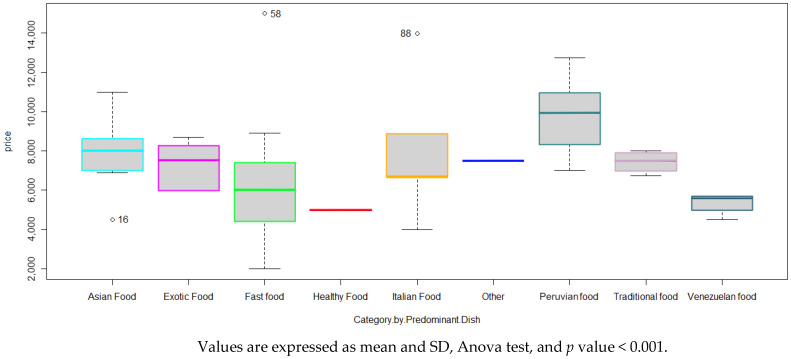
Costs of children’s menus according to categories by type of food.

Figure 5 shows the prices of the children’s menu according to restaurant category. Traditional was 7.5 (SD = 0.5), and Peruvian was 10.0 (SD = 1.7). Peruvian food presented the highest mean value and Venezuelan food restaurants the lowest mean value.

It should be noted that the healthy and other categories were excluded from this table as each had only one children’s menu and therefore only one value (healthy $5.1 and other $7.6, respectively). Significant differences were found between the mean price of children’s menus and main dish categories (*p* < 0.05) according to the Kruskal–Wallis H statistical test.

The Table 3 shows the associations between foods with the quartile with the worst score; it is observed in the raw models and in the highest-fit model that potato chips OR 40.36 (CI 95% 11.43–201.08) is the food most associated with the worst score. There are also associations with fried or processed lean meat and sweet dessert. There were no associations for other foods.

## 4. Discussion

Chile is currently in a post-transition nutritional stage; families are abandoning homemade food and opting for fast food and ultra-processed foods, following a westernization of their diet [15,26]. In addition, there is a marked preference for the consumption of high-calorie foods, with a high content of saturated fats and sugar and a decrease in the consumption of fruits and vegetables, which is in line with the results of our research.

Of the total number of restaurants analyzed in the Uber Eats application in greater Santiago, only 7% offered children’s menus, and each restaurant had between 1 and 4 menu options available. The children’s menus evaluated presented a negative score in KIMEHS, which implies an offer of “unhealthy” food, mainly due to the presence of fried and processed protein sources, chips, bread, sugary drinks, and the almost null presence of vegetables, legumes, water, and fruit, fulfilling the hypothesis stated at the beginning of the research. These results are similar to those obtained by other countries (Portugal, Croatia, Brazil) [19,22,27,28]. Since they are not nutritionally balanced and have high caloric content and peculiar non-nutritional attributes that promote their excessive consumption, ultra-processed products are most likely the main dietary cause of weight gain and prevalence of chronic diseases [15,29].

The frequent presence of these foods, in particular the most common food on menus, French fries, can have negative effects on health, as they have a significant contribution of calories from fat [30] and may contain trans fats [31]. Some studies show harmful effects on our cardiovascular health, cancer, and diabetes when included in the diet in large quantities [32]; also, the consumption of fried foods has been associated with obesity [33].

Secondly, the presence of lean meats and fried or processed meats, which are frequently present in these menus, and although the consumption of lean meats can be beneficial to health for its contribution of protein, iron, zinc, and other nutrients [34], frequent consumption, particularly in fried versions or processed meats, may contain less protein and micronutrients and more saturated fat, sodium, and other preservatives, which can have negative effects on the health of children [35,36].

In the case of sugary drinks, their frequent consumption has been linked to increased BMI [37], body weight, altered plasma lipids (total cholesterol, triglycerides, and LDL-cholesterol) [38], asthma [39], and dental caries [40]. In our study, its contribution to menus was possibly underrepresented, as it is sold separately from menus in delivery applications.

On the other hand, the food insecurity of Chilean children has increased in recent years, generating hidden hunger, a term used to describe the nutritional insufficiency of micronutrients. In this case, the current high rates of obesity could be generated by foods that provide energy but not necessarily the critical nutrients for the growth and development of students, such as iron, calcium, zinc, vitamin D, and vitamin B complex, among others [41]. This new supply of affordable and mass-market foods can replace other, more nutritious foods.

Concerning protein sources, fish appears on the menu occasionally (6%), of which approximately 50% is fried. It was also observed that available water is an option among the sugar-sweetened beverages, allowing free selection by children.

Most children’s menus did not include collectible toys since only 8% included this offer; the data are very close to those obtained in the multicenter study of Viegas et al. [19].

On the other hand, our results differ from those obtained in that study in the items sweet desserts and availability of red meat, which, although in our study is high, is surpassed by fried or processed lean meat (generally chicken/nugget). This difference could be due to the increase in the value of meat products given greater demand and shortage of supplies as a result of the pandemic, variation in the consumer price index (CPI), among others.

The availability of nutritional information was null in the children’s menus evaluated, although it was present in other menus for the general public, especially in restaurants in the healthy category or FIT (without children’s options) with accounting of macronutrients. The data are similar to those obtained in Santiago de Chile in 2015 by Ñunque et al., where in order to analyze the healthy characteristics of children’s menus, they took as a reference the compliance with the Chilean Dietary Guidelines of that time, the parameters with the worst compliance being the incorporation of legumes and nutritional information [18].

It should be noted that in 2012, a legislation was submitted to the Health Committee that “obliged commercial establishments to provide consumers with nutritional information on the food they sell, regardless of the place where they are consumed”, with the exception of the smallest ones (SMEs), Unfortunately, this motion has been shelved since 2014 [42]. This initiative sought to provide consumers with more information regarding the nutritional characteristics of the products offered in terms of the amount of energy and macronutrients (carbohydrates, proteins, and lipids) as well as sodium, specifying the type of food, with details of each basic preparation. Considering that this initiative arose 10 years ago and was based on current national statistics, we consider that if it had been implemented, it may have impacted positively on the nutritional status of our population [43]. For this reason, it is necessary to take up these shelved projects to encourage restaurants to incorporate healthier options for children, reformulating existing menu items and adding new healthier items, publishing nutritional declaration on menus and thus establishing new nutrition standards for marketing to children [26]. Taking action has already been performed in other countries such as the USA, some states in Australia, Canada, and lately in the UK [44] and Puerto Rico [45], among others.

Unexpectedly, no significant differences were observed between the cost of the children’s menus in the communes grouped by SPI. It was expected that the lower the social priority, the higher the menu’s cost. We should consider at this point that we did not accept repeated restaurant chains in the study, so one or another commune benefited from the accounting of children’s menus in these restaurants, which are generally located in several communes of the MR, unlike the KIMEHS validation study, which included repeated restaurants [46].

On the other hand, we must also consider what the menu includes; for example, in the category of communes with high social priority, the most economical children’s menu costs $7000 pesos. It corresponds to a Peruvian food restaurant and consists of a portion of French fries and 10 chicken nuggets or viennoiseries. On the other hand, in the category of communes without social priority, the cheapest children’s menu costs $4350 pesos, corresponds to a fast food restaurant, and consists of a King Jr. combo (a simple junior hamburger plus small fries and a collectible toy). In this case, we can see that the same restaurant exists in several other communes of different SPI, but in this case, it was selected in a commune without social priority, giving it the value of the cheapest menu. However, a significant difference was found between the average price of children’s menus and the main course categories, a difference from the results obtained by Viegas et al. in their multicenter study conducted in five countries [19]. This may be due to the type of restaurant included in the study, since it was carried out in shopping malls, which have a more homogeneous standard of values and premises, while ante delivery applications include all types, such as family restaurants to large fast food chains.

Another important point is the lack of information on allergens, which was found to be available in only 1% despite the fact that the Uber Eats application provides the tools to include them with the filter “options for people with allergies” and distinctive icons. The data were similar to those obtained by Ñunque et al. [18], where none of the fast food restaurants analyzed had information on allergens (special), and to those of Viegas et al. [19], where information on allergens was globally not available on the menu in the different countries studied.

Availability of children’s menus and the greatest number of restaurants in the delivery APP were in the communes located in the categories of medium low social priority to no social priority. Thus, the medium high social priority and high social priority communes presented almost no restaurants for analysis or children’s menus. This probably arises due to issues of social vulnerability, segregation at the level of services in general, purchasing power, differences in demand, and obtaining and using credit cards, among others. We should also consider that most delivery applications are associated with an extra payment for their use, which is usually concentrated in a bank card, which acts as a filter, concentrating their use in communities with greater purchasing power [46]. On the other hand, Ciper Chile, together with the Center for Research and Journalistic Projects of the UDP, conducted a report where 174 towns, villages, or neighborhoods of 31 communes of the Metropolitan Region, which are considered conflict zones under narco-domination, where for those who live in these areas, delivery is not an option, This is a portrait of the violence and lack of protection suffered by the most vulnerable sectors of the capital [47], which may explain what we found in our results, that more vulnerable sectors have less access to this type of food delivered by delivery.

Among the limitations of this study, we highlight some ambiguities found in the delivery application; for example, the reference image of the product or the detail of what is included in the menu is not always present; therefore, on certain occasions, there is no certainty that the image really corresponds to what will be received. Regarding the fruit, in the case of the options found online, it was always as fruit purees and not as natural fruit, which was given the corresponding score. Given the structure of the questionnaire, on certain occasions, a positive score is given to menus that do not include certain items in their “kids menu”, for example, potato chips, sweet desserts, or sugary drinks (the last two are generally not included in the value of the menu), but this does not mean that they do not buy or consume them anyway. The sample size and geographic location of the sample also impair generalization of the results to other regions of Chile, giving rise to future research that can be replicated in other regions/communes of our country and using different APPs. This study also has some important strengths to mention, such as the use of a survey that has been validated to measure menus for children and that has been used in several countries around the world, as well as the fact that this study included a large number of communes in the metropolitan region, which includes 3 million people. For future research, it is necessary to be able to use, in addition to this survey, information on the prevalence of childhood obesity in the city or area, to be able to analyze the food environments or measure the density of supply in order to continue exploring this emerging area, as well as to carry out dietary analysis with the aim of determining the contribution in calories and nutrients of this type of food in children.

The findings of the present study are a contribution to the validation and applicability of the tool used since they provide acceptable data on the reliability of the questionnaire, which are not far from the data previously obtained by other authors in Chile. Evidently, there is still a long way to go in terms of obtaining a greater number of data for analysis, considering the different delivery platforms currently available. There are no Chilean studies that evaluate in depth the available children’s menus, which is a niche of research opportunities considering that we already have a valid and reliable instrument to estimate the valuation of a menu in order to generate more scientific evidence that allows us to implement intervention strategies in large and small food chains in favor of children’s health new marketing strategies oriented to a healthy eating behavior, focused according to age, gender, activity, and social priority.

## 5. Conclusions

By analyzing the results, the hypothesis that the offer of children’s menus offered in fast food restaurants present in delivery applications in different communes of the city of Santiago is not considered healthy has been proven, in particular by the regular presence of chips and processed meats. It is concluded that most of the restaurants offer a children’s menu of low nutritional quality and poor balance, including mainly dishes based on fried and processed products. It is urgent to legislate on the quality of food delivered to children’s menus using app delivery as they are becoming increasingly popular, inexpensive, and very accessible.

## Figures and Tables

**Table 1:** Availability (%) of KIMEHS components in the evaluated menus.

Food Items	% Availability
Red meat	27.1 (31)
Lean meat	64.9 (74)
Fried red meat	18.4 (21)
Processed red meat	18.4 (21)
Fried or processed lean meat	42.1 (48)
Fish	6.1 (7)
Fried fish	3.5 (4)
Pulses	0.8 (1)
Chips	71.0 (81)
Bread, rice, and pasta	50.8 (58)
Vegetables	
Non-starchy vegetables	21.0 (24)
Soup	0 (0)
Dessert	
Fruit	5.2 (6)
Sweet dessert	6.1 (7)
Beverage products	
Water	3.5 (4)
Sugary beverages	15.7 (18)
Allergen info	1.7 (2)
Nutritional info	0 (0)

**Table 2:** Availability (%) of protein sources in children’s menus according to category by type of meal.

Food		Restaurant Typology								
		Asian Food	Fast Food	Exotic Food	Italian Food	Peruvian Food	Traditional Food	Venezuelan Food	Healthy Food	Other
Protein sources	Red meat	9.1 (2)	50 (17)	20 (1)	20 (1)	10.7 (3)	28.6 (4)	50 (2)	100 (1)	0
	White meat	77.3 (17)	50 (17)	80 (4)	80 (4)	71.4 (20)	57.1 (8)	50 (2)	100 (1)	100 (1)
	Fried red meat	0	47.1 (16)	0	20 (1)	0	7.1 (1)	50 (2)	0	0
	Processed red meat	0	47.1 (16)	0	20 (1)	0	14.2 (2)	50 (2)	0	0
	Fried or processed meat	40.9 (9)	44.4 (15)	60 (3)	40 (2)	39.3 (11)	35.7 (5)	50 (2)	0	100 (1)
	Fish	13.6 (3)	0	0	0	14.3 (4)	0	0	0	0
	Fried fish	9.1 (2)	0	0	0	3.6 (1)	0	0	0	0
	Pulses	0	0	0	0	0	0	0	100 (1)	0
Chips, bread, rice, and pasta	Chips	50 (11)	82.4 (28)	100 (5)	40 (2)	82.1 (23)	50 (7)	100 (4)	0	100 (1)
	Bread, rice, and pasta	63.6 (14)	58.8 (20)	40 (2)	40 (2)	28.6 (8)	71.4 (10)	50 (2)	100 (1)	0
Vegetables	Vegetable	27.3 (6)	14.7 (5)	40 (2)	60 (3)	10.7 (3)	28.6 (4)	0	100 (1)	0
	Soup	0	0	0	0	0	0	0	0	0
Dessert	Fruit	0	14.7 (5)	0	0	3.6 (1)	0	0	0	0
	Sweet dessert	13.6 (3)	2.9 (1)	0	0	3.6 (1)	14.2 (2)	0	0	0
Beverage products	Water	0	8.8 (3)	20 (1)	0	0	0	0	0	0
	Sugary beverages	13.6 (3)	17.6 (6)	20 (1)	20 (1)	10.7 (3)	35.7 (5)	0	0	0

**Table 3:** Association of less healthy menus and unhealthy foods.

Unhealthy Foods	Model 1 OR (CI 95%)	Model 2 OR (CI 95%)	Model 3 OR (CI 95%)
Chips	20.63 (7.54–65.04) ***	18.86 (6.81–59.93) ***	40.36 (11.43–201.08) ***
Fried or processed lean meat	2.03 (0.75–5.79)	2.34 (0.79–7.14)	3.90 (1.16–14.56) *
Sugary beverages			1.95 (0.38–12.65)
Sweet dessert			35.66 (2.66–1051.68) *
Fried fish			0.38 (0.02–5.27)

Signif. codes: ‘***’: <0.001; ‘*’: <0.05. Model 1: The two unhealthy foods most present on children’s menus. Model 2: the two unhealthy foods present in third and fourth place on children’s menus. Model 3: all the unhealthy foods present on children’s menus.

## Data Availability

The original contributions presented in the study are included in the article/Supplementary Materials, further inquiries can be directed to the corresponding author.

## Supplementary Materials

The following supporting information can be downloaded at: https://www.mdpi.com/article/10.3390/foods14030434/s1, Table S1: Communes of the MR according to their SPI category that presented children’s menu options; Table S2: Step-by-step to make a purchase through the Uber Eats app.
