# Supplementary material for: Children’s Menus at Fast Food Restaurants on the Uber Eats® Delivery App
Source: Foods. 2025 Jan 29;14(3):434. doi: 10.3390/foods14030434 (PMC11817455; doi:10.3390/foods14030434)
Supplement: Supplementary file 1 [file foods-14-00434-s001.zip › foods-3351058-supplementary.pdf]

Supplementary Table S1: Communes of the MR according to their SPI category that presented children's menu options

SPI category	Communes
**High Social Priority**	San Ramon
**Medium-high Social Priority**	San Bernardo
	San Joaquín
	Renca
**Medium-low Social Priority**	Puente Alto
	Peñalolén
	La Cisterna
	Independencia
**Low Social Priority**	Maipú
	San Miguel
	Huechuraba
	Santiago
	Macul
**Without Social Priority**	Ñuñoa
	Las Condes
	Providencia
	La Reina
	Vitacura

Supplementary Table S2: Step-by-step to make a purchase through the Uber Eats app

1. Login to the application from your mobile phone or computer (www.ubereats.com).

2. Enter the address where you want to receive the order.

3. On the main page different options appear such as sushi, pizza, hamburgers, Chinese food, among others. In addition, you can select filters such as ‘delivery in less than 30 minutes’, ‘cost’, ‘shipping cost’, ‘type of diet’, etc. Also appearing on this main page are the sections ‘today's specials’, ‘nearby shops’, ‘most popular local restaurants’, ‘only on Uber Eats’, among others.

4. Select the restaurant, in this case McDonald's. The nearest store to the address entered will appear, with important information such as delivery cost, approximate delivery time and featured items (products, combos).

5. Select ‘Happy Meal’, which corresponds to the children's menu in this restaurant. The available varieties of Happy Meal will appear, with images of the products, components and value.

6. Return to the McDonald's homepage where you can continue to add products or ‘combos’ to your order.

7. Go to the top right hand side of the screen and select ‘Our cart’ where the Happy Meal and its value will appear. Click on ‘Continue’ and, if you have already created an account, you will see the delivery details such as: address (modifiable), payment method (modifiable), cart summary (modifiable) and details of the order cost.

8. Once you have checked all the details, select ‘Place the order’ and your production will start in the shop to later receive it at your home.

Step-by-step to make a purchase through the Uber Eats app (with images)

1. Login to the application from your mobile phone or computer (www.ubereats.com).

2. Enter the address where you want to receive the order.

3. On the main page different options appear such as sushi, pizza, hamburgers, Chinese food, among others. In addition, you can select filters such as ‘delivery in less than 30 minutes’, ‘cost’, ‘shipping cost’, ‘type of diet’, etc. Also appearing on this main page are the sections ‘today's specials’, ‘nearby shops’, ‘most popular local restaurants’, ‘only on Uber Eats’, among others.

   1. Select the restaurant, in this case McDonald's.

   2. The nearest store to the address entered will appear, with important information such as shipping cost, approximate delivery time and featured items (products, combos).

3. Select ‘Happy Meal’, which corresponds to the children's menu in this restaurant. The available varieties of Happy Meal will be displayed, with images of the products, components and value.
4. Select the Happy Meal you wish to purchase, in this case, the ‘Junior Party Happy Meal’.
5. A new window will appear where you will have to choose the mandatory fields which are the ‘drink’ (options of water, small juice or Coca-Cola, Fanta or sugar-free Sprite) and the ‘toy’ (options of book or surprise toy) and the quantity. This Happy Meal option also includes a compote and 6 units of duchess potatoes (not modifiable).
6. Once ready, select ‘Add to order’.
7. With the information available on the platform it is possible to carry out the evaluation of the available children's menus.
